# Bioflocculants’ production in a biomass-degrading bacterium using untreated corn stover as carbon source and use of bioflocculants for microalgae harvest

**DOI:** 10.1186/s13068-017-0987-6

**Published:** 2017-12-20

**Authors:** Haipeng Guo, Chuntao Hong, Bingsong Zheng, Fan Lu, Dean Jiang, Wensheng Qin

**Affiliations:** 1 Department of Biology, Lakehead University, Thunder Bay, ON P7B 5E1 Canada; 2 State Key Laboratory of Plant Physiology and Biochemistry, College of Life Sciences, Zhejiang University, Hangzhou, 310058 China; 3 Academy of Agricultural Sciences of Ningbo City, Ningbo, 315040 China; 4 State Key Laboratory of Subtropical Silviculture, Zhejiang A & F University, Hangzhou, 311300 China; 5 School of Biological Engineering, Hubei University of Technology, Wuhan, 430068 China

**Keywords:** Biomass-degrading bacterium, *Pseudomonas* sp. GO2, Corn stover, Bioflocculants, Microalgae harvest

## Abstract

**Background:**

Bioflocculation has been developed as a cost-effective and environment-friendly method to harvest multiple microalgae. However, the high production cost of bioflocculants makes it difficult to scale up. In the current study, low-cost bioflocculants were produced from untreated corn stover by a biomass-degrading bacterium *Pseudomonas* sp. GO2.

**Results:**

*Pseudomonas* sp. GO2 showed excellent production ability of bioflocculants through directly hydrolyzing various biomasses. The untreated corn stover was selected as carbon source for bioflocculants’ production due to its highest flocculating efficiency compared to that when using other biomasses as carbon source. The effects of fermentation parameters on bioflocculants’ production were optimized via response surface methodology. According to the optimal model, an ideal flocculating efficiency of 99.8% was obtained with the fermentation time of 130.46 h, initial pH of 7.46, and biomass content of 0.64%. The relative importance of carboxymethyl cellulase and xylanase accounted for 51.8% in the process of bioflocculants’ production by boosted regression tree analysis, further indicating that the bioflocculants were mainly from the hydrolysates of biomass. Biochemical analysis showed that it contained 59.0% polysaccharides with uronic acid (34.2%), 32.1% protein, and 6.1% nucleic acid in the bioflocculants, which had an average molecular weight as 1.33 × 10^6^ Da. In addition, the bioflocculants showed the highest flocculating efficiency at a concentration of 12.5 mg L^−1^ and were stable over broad ranges of pH and temperature. The highest flocculating efficiencies obtained for *Chlorella zofingiensis* and *Neochloris oleoabundans* were 77.9 and 88.9%, respectively.

**Conclusions:**

The results indicated that *Pseudomonas* sp. GO2 can directly utilize various untreated lignocellulolytic biomasses to produce low-cost bioflocculants, which showed the high efficiency to harvest two green microalgae in a low GO2 fermentation broth/algal culture ratio.

**Electronic supplementary material:**

The online version of this article (10.1186/s13068-017-0987-6) contains supplementary material, which is available to authorized users.

## Background

Owing to the enormous demand for energy and the shortage of traditional fossil fuels, renewable and sustainable fuels have received increasing attention to alleviate dependence on the fossil fuels. Microalgae have been considered as promising renewable feedstocks owing to their fast growth rate, high lipid accumulation as well as non-competition with food supply [1, 2]. Although biofuel production from microalgae has been widely researched in the last few decades, it is still hindered by high production costs, which mainly comes from the processes of cultivation, harvest, drying, lipid extraction, and transesterification [3, 4]. The harvest of algae, which usually accounts for more than 30% of total costs, has been one of the biggest challenges in the process of algal biofuel production [5, 6]. Furthermore, costs may increase according to the size of microalgal cells, the density of the algal culture, and the intensity of negative charge on the surface of algae [7]. Therefore, an economic and effective method is urgently necessary for algal harvesting to reduce total costs [5].

A variety of methods, such as centrifugation [8], filtration [9], ultrafiltration [10], sedimentation [5], and flotation [11], have been used to harvest algal cells. However, high-capital equipment and operational costs, high energy consumption, or heavy dependence on algal species have hampered large-scale commercialization of these microalgal harvesting methods [5, 12]. The harvest of microalgae through flocculation, which can form heavy aggregates by neutralizing the algal surface’s negative charges using various flocculants and thus increase the rate of settling [13], has been proposed as the most reliable and suitable harvesting method for multiple algae species, even at low biomass concentrations [6, 14]. Flocculation induced by chemicals, including inorganic and organic chemicals, has been used for the effective harvest of various microalgal strains. However, inorganic flocculants are toxic and have negative effects on algal viability, thus limiting algal recycling and reuse [15]. Moreover, most organic flocculants are mainly derived from fossil fuels or edible crops, which may further aggravate the problems of fossil fuel and food shortages, and associated environmental risks [5, 16].

Bioflocculants’ production by bacteria in nature has been suggested as a cost-effective and environment-friendly method of producing flocculants to effectively harvest multiple algae [17, 18]. These bioflocculants mainly consisted of polysaccharides, proteins, and nucleic acids [19]. Some bacterial species like *Bacillus* sp. [20], *Rhodococcus* sp. [21], *Solibacillus* sp. [22], *Arthrobacter* sp. [23], and *Pseudomonas* sp. [24] have been reported to produce bioflocculants, and applied to harvest algae. However, for commercial-scale bioflocculants’ production from bacteria, large amounts of nutrients and sugars are required [18, 24]. To minimize the production cost, various wastewaters and hydrolysates of agricultural waters have been successfully used for bacterial growth and bioflocculants’ production [24, 25]. Recently, some lignocellulolytic enzyme-producing bacteria have been found to possess the ability to produce bioflocculants through directly utilizing untreated renewable lignocellulosic biomass. Liu et al. [18] reported that *Cellulosimicrobium cellulans* L804 can produce bioflocculants from untreated corn stover by secreting CMCase and xylanase. Bioflocculants produced by *Bacillus agaradhaerens* C9 using untreated rice bran have been shown to be highly efficient in harvesting algae [26]. Here, a novel biomass-degrading bacterium isolated from paper mill sludge showed efficient bioflocculants’ production using various untreated lignocellulosic biomasses as carbon source. Then, the bioflocculants’ production conditions were further optimized via response surface methodology (RSM) and evaluated by boosted regression tree (BRT) analysis. In addition, their flocculating properties and applications in two types of microalgal harvesting were also evaluated in this study.

## Results and discussion

### Evaluating the biomass degradation ability of GO2

**Fig. 1:**
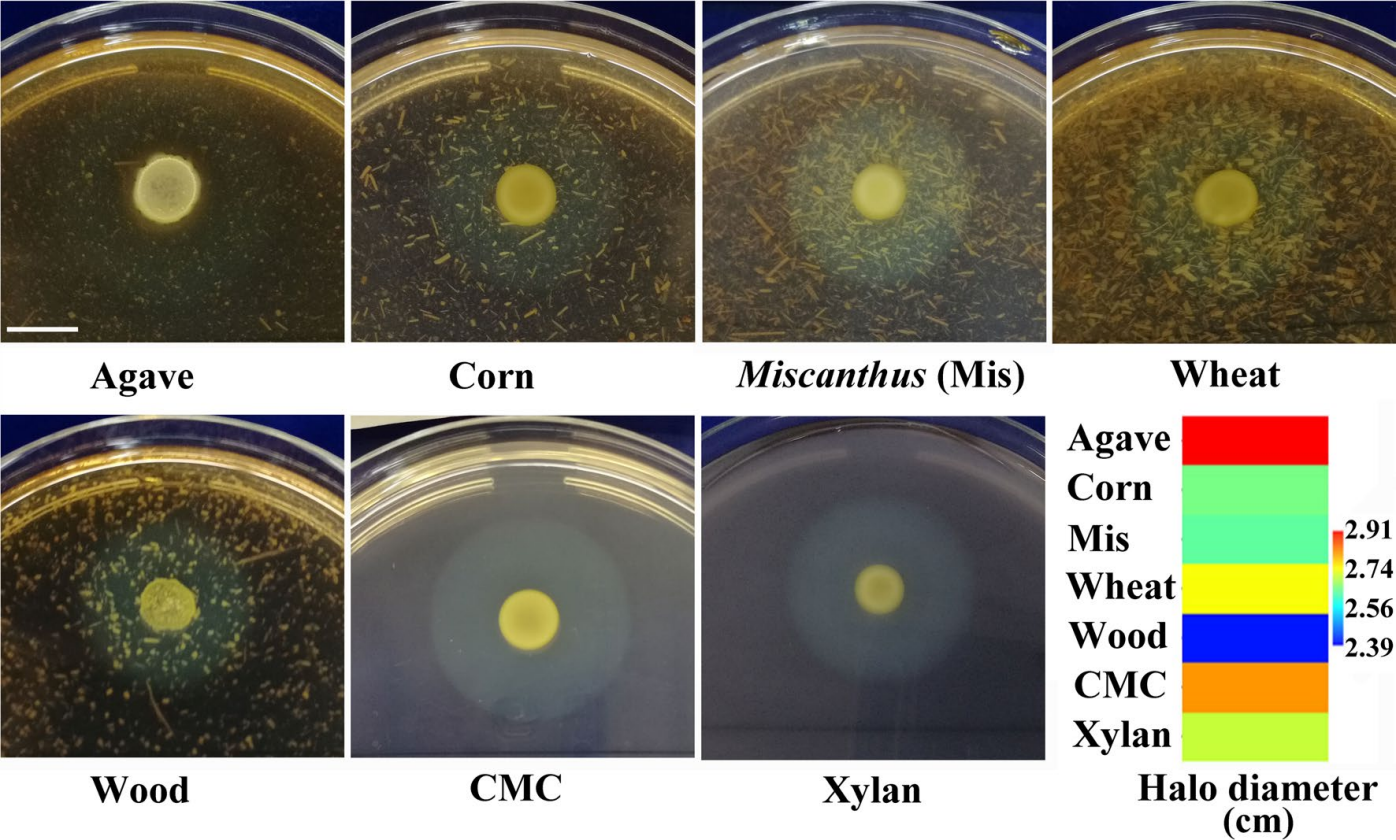
Evaluation of the hydrolysis ability of *Pseudomonas* sp. GO2 using different biomass, CMC, or xylan as carbon source by Gram’s iodine staining. The yellow plaque in the center of plate indicates the bacterial size, and the halo region indicates that carbohydrate polymers are hydrolyzed. The halo diameter is shown by matrix plot. Bar = 1 cm

One isolate, GO2, which could produce bioflocculants by directly degrading various untreated biomasses, was isolated from paper mill sludge and finally identified as *Pseudomonas* sp. according to the morphological and phylogenetic characteristics of this strain, as well as the sequence of 16S rRNA (Additional file 1: Fig. S1). The sequence has been submitted to NCBI (https://www.ncbi.nlm.nih.gov/), and the accession number was given as MF448527. The biomass hydrolysis ability of this strain was evaluated by Gram’s iodine staining on plates using different lignocellulolytic biomasses as the sole carbon source (Fig. 1). Gram’s iodine staining has been regarded as an economic, fast, and environmentally friendly qualitative method for evaluating the hydrolysis ability of microorganisms on an agar plate [27]. Gram’s iodine forms a bluish-black compound with carbohydrate polymers but not with their hydrolysates (Halo region). In this study, the strain GO2 produced an obvious halo region with the halo diameters ranging from 2.4 to 2.9 cm in the agar plate containing untreated agave, corn stover, *Miscanthus*, wheat bran, and wood dust (Fig. 1). In addition, similar halo regions can be formed with the halo diameters of 2.8 and 2.7 cm in the agar plates containing CMC and xylan, respectively, indicating the strain GO2 was capable of degrading the biomass through secreting CMCase and xylanase [28, 29]. A number of *Pseudomonas* strains have been reported to produce various extracellular lignocellulolytic enzymes, which showed excellent ability to hydrolyze various lignocellulosic feedstocks [30, 31].

### Selecting the best lignocellulose biomass for bioflocculants’ production

**Fig. 2:**
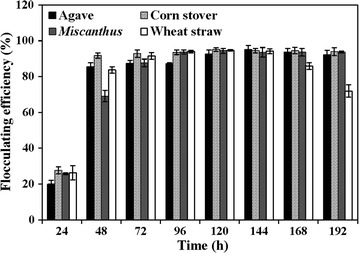
Bioflocculants’ production by *Pseudomonas* sp. GO2 using 0.5% untreated agave, corn stover, *Miscanthus,* or wheat bran biomass as carbon source. Strain GO2 was cultured at 37 °C and 200 rpm for 8 days, and the supernatants were collected each day to measure the flocculating efficiency by means of kaolin clay as substrate. Data are the means of four replicates ± SE

Lignocellulose biomass, which is mainly made up of complex carbohydrate polymers including cellulose, hemicellulose, and lignin, has been widely used for the production of biofuels and other biomass-derived value-added chemicals and bioproducts [32, 33]. In this study, four main lignocellulose biomasses including agave, corn stover, *Miscanthus*, and wheat bran were selected to evaluate the bioflocculants’ production ability using the biomass-degrading strain *Pseudomonas* sp. GO2. The maximum flocculating efficiency with the value of 95.1% was obtained using 0.5% (w/v) corn stover after 120 h of fermentation, while the highest flocculating efficiencies were 95.0, 94.4, and 94.7% in the presence of 0.5% (w/v) agave, *Miscanthus*, and wheat bran, respectively, after 120–144 h of incubation (Fig. 2). Moreover, the maximum flocculating efficiency was only 23.5% after 48 h of fermentation in the absence of biomass (Data not shown), representing that the bioflocculant was primarily produced from the hydrolysates of lignocellulose biomass by *Pseudomonas* sp. GO2. The flocculating efficiency of GO2 induced by 0.5% untreated corn stover was almost always higher and more stable than that by other biomasses after 48–196 h of incubation (Fig. 2), and its maximum flocculating efficiency (95.1%) was significantly higher than that of *Cellulosimicrobium cellulans* L804 induced by 2.0% corn stover (90.9%) [18] and *Bacillus agaradhaerens* C9 produced by rice bran (87.2%) [26]. Therefore, untreated corn stover was selected as the best lignocellulose biomass candidate for bioflocculants’ production in the subsequent experiments.

### Optimization of the bioflocculants’ production using GO2

The effects of incubation time, biomass content, and initial pH on flocculating efficiency of GO2 fermentation were optimized by RSM. Variance analysis (ANOVA) suggested that the model was significant because the *F* value of the model was 5.46, which stands for only a 0.7% probability to come up because of noise (Table 1). The correlation coefficient (*R* ^2^) was recorded up to 0.9390, which represented that the correlation of experimental and predicated values was significant in the model [21]. Moreover, the low “Pro > *F*” value (< 0.0500) demonstrated that the model terms were significant (Table 1). According to the optimal model, the fitted equation of flocculating efficiency during fermentation process is finally shown as follows:

$$\begin{aligned} {\text{Flocculating activity }} & = -\,97.74941 + 0.87408\,\left[ {\text{Time}} \right] + 49.61787\,\left[ {\text{Initial pH}} \right] \\ & \quad - 41.92902 \,\left[ {\text{Biomass content}} \right] - 0.059458 \, \left[ {\text{Time}} \right] \times \left[ {\text{Initial pH}} \right] \\ & \quad + 0.028872 \, \left[ {\text{Time}} \right] \times \left[ {\text{Biomass content}} \right] + 0.47619\,\left[ {\text{Initial pH}} \right] \\ & \quad \times \left[ {\text{Biomass content}} \right] - 1.28178 {\text{E}}{-}003\,\left[ {\text{Time}} \right]^{ 2} \\ & \quad - 3.35267\,\left[ {\text{Initial pH}} \right]^{ 2} + 6.03965\,\left[ {\text{Biomass content}} \right]^{ 2} \\ \end{aligned}$$

**Table 1:** ANOVA of the quadratic model coefficient of flocculating efficiency

Source	Sum of squares	*DF*	Mean square	*F* value	Prob > *F*
Model	8719.77	9	968.86	5.46	0.0070
*A*	2433.14	1	2433.14	13.72	0.0041
*B*	61.99	1	61.99	0.35	0.5675
*C*	2599.69	1	2599.69	14.66	0.0033
AB	197.34	1	197.34	1.11	0.3164
AC	34.71	1	34.71	0.2	0.6676
BC	17.72	1	17.72	0.100	0.7584
*A* ^2^	54.69	1	54.69	0.31	0.5909
*B* ^2^	640.74	1	640.74	3.61	0.0865
*C* ^2^	1203.5	1	1203.5	6.78	0.0263
Residual	1773.86	10	1773.86	–	–

*R* ^2^ 0.9390, *A* fermentation time (h), *B* initial pH, *C* biomass content (%), *DF* degree of freedom

**Fig. 3:**
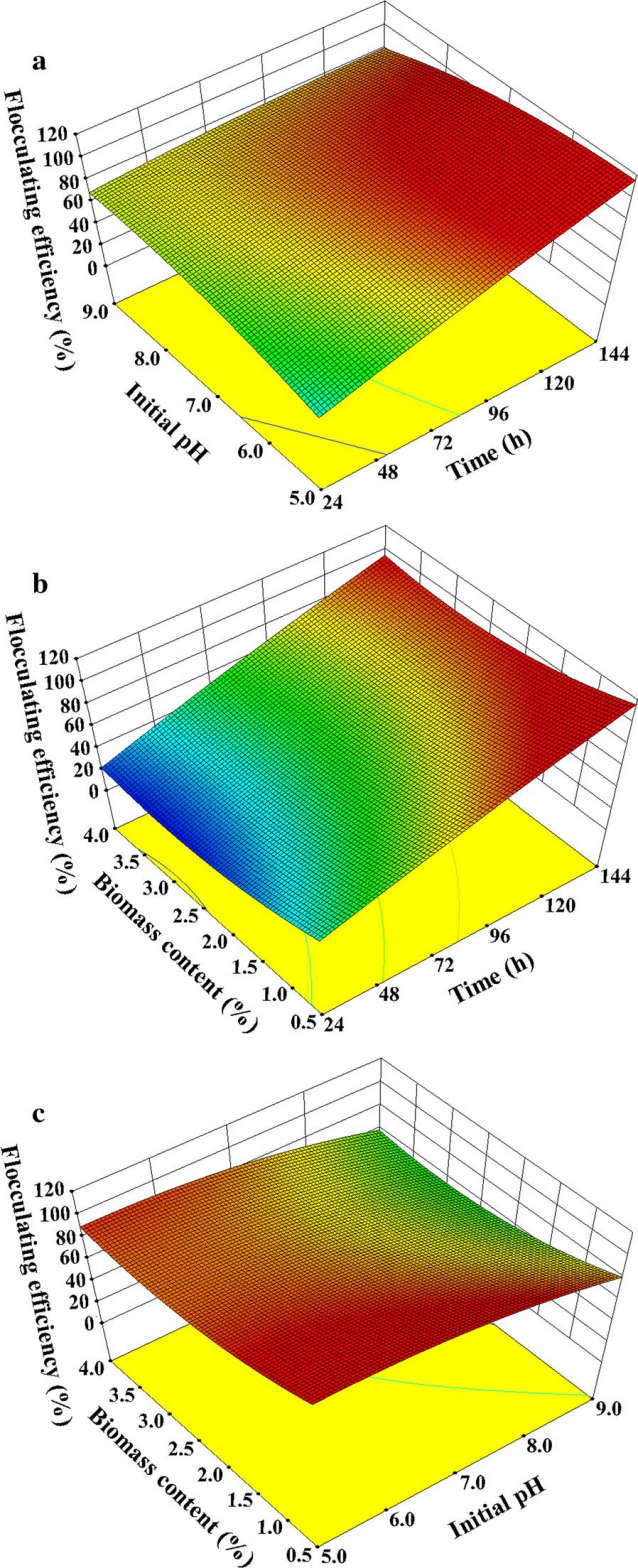
Effects of fermentation time, initial pH, and biomass content on bioflocculants’ production using *Pseudomonas* sp. GO2 via RSM. The matrix included a total of 20 experiments, and all experiments were performed at 37 °C and 200 rpm. **a** Effects of fermentation time and initial pH. **b** Effects of fermentation time and biomass content. **c** Effects of initial pH and biomass content

As shown in Fig. 3, the flocculating efficiency can achieve an ideal level of 99.8% under the optimal conditions of fermentation time of 130.46 h, initial pH at 7.46, and biomass content at 0.64% (w/v). The flocculating efficiency was positively related to incubation time, but decreased slightly with further extension of culture time, possibly due to the limited growth of cells caused by consumption of various nutrients [34] and the production of proteases caused by cell lysis [35]. Moreover, it seemed that the biomass content negatively affected the increase of flocculating efficiency, and the initial pH only showed a little influence on the bioflocculant production (Fig. 3). The high biomass content may markedly change the ratio of carbon and nitrogen, which played a crucial role in cell growth and bioflocculant production [21, 26]. High carbon/nitrogen ratio is beneficial to cell growth but decreases flocculating efficiency and the accumulation of bioflocculants. The low carbon/nitrogen ratio can maintain a stable pH of the culture medium, thus keeping an excellent flocculating efficiency [36, 37]. The excessive biomass content may refine oxygen transfer in the cultural medium, which affected the cell growth, enzymatic secretion, and bioflocculants’ production of bacteria [26]. In addition, excess nutrition caused by high biomass contents also leads to the decrease of flocculating efficiency [21].

**Fig. 4:**
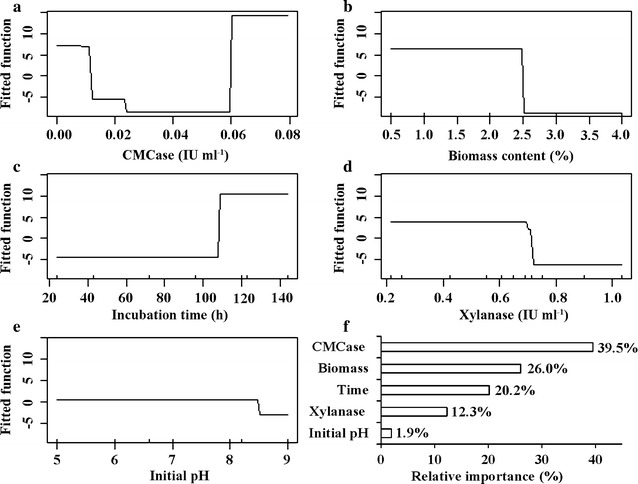
Partial dependence plots of the five predictor variables in the BRT model for bioflocculant production and their relative importance. **a** CMCase activity; **b** biomass content; **c** incubation time; **d** xylanase activity; **e** initial pH; **f** relative importance of the explanatory variables. Rug plots at the inside bottom of graph showed distribution of sample sites along that variable

To understand the relative importance of incubation time, CMCase activity, xylanase activity, biomass content, and initial pH on flocculating efficiency, a BRT modeling analysis, which combines with statistical and machine learning techniques [38], was performed. The selected model was fitted when learning rate was set as 0.001, tree complexity as 4, and bag fraction as 1.5. The model showed a training data correlation coefficient of 0.82 and a cross-validation correlation coefficient of 0.75, indicating that this model is significant [39, 40]. The relative importance analysis indicated that CMCase activity was the most important variable on flocculating efficiency (39.5%), followed by biomass content (26.0%), incubation time (20.2%), xylanase activity (12.3%), and initial pH (1.9%) (Fig. 4). The relative importance of CMCase and xylanase activity accounted for 51.8% of all variables, further indicating that the bioflocculants’ production of GO2 was mainly from the conversion of biomass by lignocellulosic enzymes [26]. The dependence plot of biomass content showed that flocculating efficiency was positively related when the biomass content was less than 2.5%, and the biomass content, which ranged from 2.5 to 4.0%, made almost no contribution to bioflocculants’ production (Fig. 4b). This was similar to that the low biomass content was beneficial to produce bioflocculants according to the RSM model (Fig. 3b). The flocculating efficiency was significantly increased after 108 h of incubation, which may mainly depend on the production of lignocellulosic enzymes and thereby release available substrates to produce bioflocculants [18, 41]. In addition, the model indicated that the optimal initial pH for flocculating efficiency ranged from 5.0 to 8.3, which is consistent with the initial pH close to neutralization leading to the highest flocculating rate [24, 42].

### Characteristics of the bioflocculants produced by GO2

The bioflocculants from GO2 consisted of 59.0% total polysaccharides, 32.1% protein, and 6.1% nucleic acid (Table 2). Our result was similar to the main compositions of bioflocculants secreted by other bacterial strains [18, 43]. Further analysis indicated that total polysaccharides contained 58.0% uronic acid. The higher contents of proteins and uronic acid in the bioflocculants can offer more carboxyl groups, which can increase the number of effective sites for particles’ adsorption [18, 44]. The maximum bioflocculants yield was 316 mg g^−1^ biomass when untreated corn stover was added into the medium. The viscosity of 1.0 g L^−1^ bioflocculants is 1.352 cPs, and the average molecular weight of these bioflocculants is 1.33 × 10^6^ Da. In addition, the bioflocculants showed a negative zeta potential with the value of − 57.38 mV, and its charge density is − 1.007 meq g^−1^ bioflocculants (Table 2).

**Table 2:** The compositions and characteristics of the bioflocculants produced from 0.5% (w/v) untreated corn stover by *Pseudomonas* sp. GO2

Compositions	
Polysaccharide	59.0 ± 3.84%
Protein	32.1 ± 2.61%
Nucleic acid	6.1 ± 0.90%
Uronic acid	34.2 ± 1.85%
Characteristics	
Maximum yield	316 ± 10.8 mg g^−1^ dry biomass
Viscosity	1.352 ± 0.008 cPs (1.0 g L^−1^)
Molecular weight	1.33 × 10^6^ Da
Charge density	− 1.007 ± 0.016 meq g^−1^ bioflocculants
Zeta potential	− 57.38 ± 0.18 mV

To further verify that the functional groups existed in the bioflocculants, the spectra of the purified bioflocculants were measured in the 4000–600 cm^−1^ region (Additional file 1: Fig. S2). A big absorption band was found at 3275 cm^−1^, indicating the existence of hydroxyl and amine groups, and a weak C–H stretching of methyl, methylene, or methane group at 2928 cm^−1^ [45]. The absorption signals at 1630 and 1450 cm^−1^ are the typical characteristics of carboxyl groups, representing the presence of proteins and amino-sugars [13]. The absorption peak at 1045 cm^−1^ represents a C–O–C stretching vibration of ester linkage, and the weak peak at 881 cm^−1^ means β-glycosidic linkages between the sugar monomers, and it has been reported that the wave numbers between 1200 and 800 cm^−1^ are the main features of all sugar derivatives [46]. The results further confirmed that the bioflocculants predominantly consist of polysaccharides and proteins.

### Effects of dosage, pH, temperature, and metal ions on flocculating activity

**Fig. 5:**
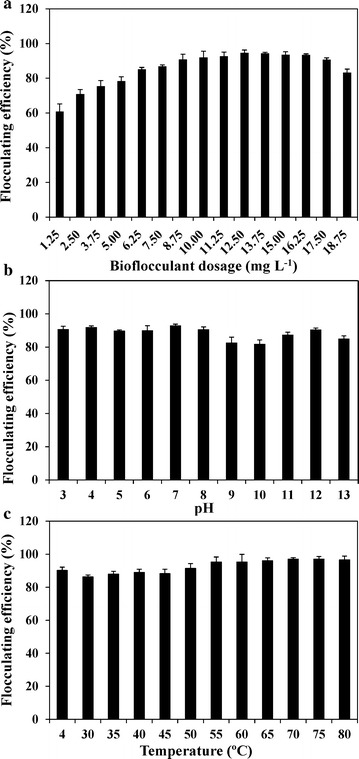
Effects of bioflocculant dosage, pH, and temperature on flocculating efficiency using kaolin clay suspension. **a** Dosage; **b** pH; **c** temperature. Data are the means of four replicates ± SE

**Fig. 6:**
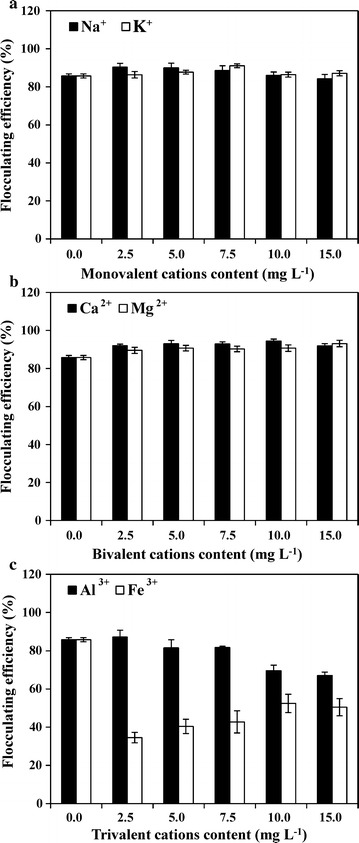
Effects of various metal ions (0–15 mg L^−1^) on flocculating efficiency using kaolin clay suspension at pH 7.0 with the bioflocculant dosage of 12.5 mg L^−1^. **a** Monovalent cations (Na^+^ and K^+^); **b** bivalent cations (Ca^2+^ and Mg^2+^), and **c** trivalent cations (Al^3+^ and Fe^3+^). Data are the means of four replicates ± SE

To determine the lowest amount of bioflocculants with the highest flocculating efficiency, a series of dosage concentrations ranging from 1.25 to 18.75 mg L^−1^ were selected to estimate the flocculating efficiency. The results showed the highest flocculating efficiency (94.7%) was obtained with a dosage of 12.5 mg L^−1^, and dosage concentrations of 8.75–17.5 mg L^−1^ resulted in more than 90% flocculating efficiency (Fig. 5a). Low dosage of bioflocculants usually led to inadequate bridging between particles, while higher bioflocculants concentrations increased the repulsion between particles because of the excessive import of negatively charged polysaccharides [18]. In addition, high dosage of bioflocculants caused the increase in the viscosity of solution, which can also inhibit the sedimentation of floccules [13]. The extracted bioflocculants showed an extensive pH range for flocculating kaolin clay (Fig. 5b). All measured pH values (3–13) achieved over 80% flocculating efficiency. pH values below 8 achieved more than 90% flocculating efficiency (Fig. 5b). pH affected the electronic states of the bioflocculants, thus influencing the flocculation efficiency [47]. The slight decrease in flocculation efficiency at pH 9 and 10 may be attributed to the weakening of the spatial charge arrangements of these bioflocculants under these pH conditions [43]. Moreover, the GO2 bioflocculants exhibited excellent characteristics of thermostability, with over 85% flocculating efficiency at all tested temperatures (4–80 °C). These are consistent with most polysaccharide-dependent bioflocculants being more stable than protein- and nucleic acid-dependent ones [48, 49]. The superior pH and temperature tolerance of these bioflocculants indicated they could have a wide variety of industrial uses regardless of the pH and temperature of the solutions, thus making them cost effective [43]. Furthermore, the flocculating efficiency was significantly enhanced by monovalent cations (Na^+^ and K^+^), bivalent cations (Ca^2+^ and Mg^2+^), and low concentration Al^3+^, while it was markedly inhibited by high concentration Al^3+^ and all tested Fe^3+^ concentrations (Fig. 6). Metal cations can stabilize and neutralize the residual negative charge formed by certain functional groups, and help to form the bridges between particles, thus improving flocculating efficiency [50]. The highest flocculating efficiency was obtained using 10 mg L^−1^ Ca^2+^ (Fig. 6b), which has been found to be the best cation stimulator for most bioflocculants [18, 19]. The poor flocculation in the presence of Fe^3+^ (Fig. 6c) may be due to the alteration of the surface charge and the decrease in adsorption sites of particles [43].

### Application of the bioflocculants GO2 in two green microalgal harvests

**Fig. 7:**
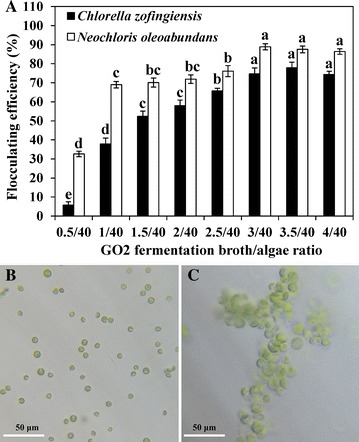
**A** Effects of different volume ratios of GO2 fermentation broth/algal culture on the flocculating efficiencies of two green microalgae, *Chlorella zofingiensis* and *Neochloris oleoabundans*. Microscopic views of *Neochloris oleoabundans* cells before (**B**) and after (**C**) bioflocculation. Data are the means of four replicates ± SE, and bars with different letters are significantly different at *p* < 0.05 according to Duncan’s multiple range test. Bar = 50 μm

Green microalgae have been regarded as an emerging feedstock to produce biodiesel, but the commercialization of microalgal biofuel production has not been realized because of its high production cost [51]. In this study, a cost-effective algal harvesting method using bioflocculants produced by *Pseudomonas* sp. GO2 was investigated. To save as much cost as possible, the fermentation broth of GO2 was directly mixed with algal culture in different volume ratios to evaluate its flocculating efficiency. The results showed the flocculating efficiencies of two green microalgae were significantly enhanced by increasing the ratio of GO2 fermentation broth and algal culture (Fig. 7A). The maximum flocculating efficiency was 77.9% at the GO2 fermentation broth/algal culture ratio of 3.5/40 for *Chlorella zofingiensis* (*C. zofingiensis*), while it was up to 88.9% for *Neochloris oleoabundans* (*N. oleoabundans*) at the corresponding ratio of 3/40 (Fig. 7A). The dosage of GO2 bioflocculants was significantly lower than that of other bacterial bioflocculants. *Cellulosimicrobium cellulans* L804 started to show the effective flocculating ability only when the fermentation broth and algal culture ratio was up to 1/6 for flocculating *Chlamydomonas reinhardtii* and 1/2 for flocculating *Chlorella minutissima* [18]. The corresponding ratio of 3/1 was needed by using the fermentation broth of *Solibacillus silvestris* W01 although 90% microalga *Nannochloropsis oceanica* can be harvested [22]. Moreover, the images showed that the algal cells were separately scattered before flocculation (Fig. 7B), while they were connected as large aggregates after flocculation (Fig. 7C), thus settling easily due to faster sedimentation [13].

### The possible mechanism of flocculation for harvesting microalgae

**Fig. 8:**
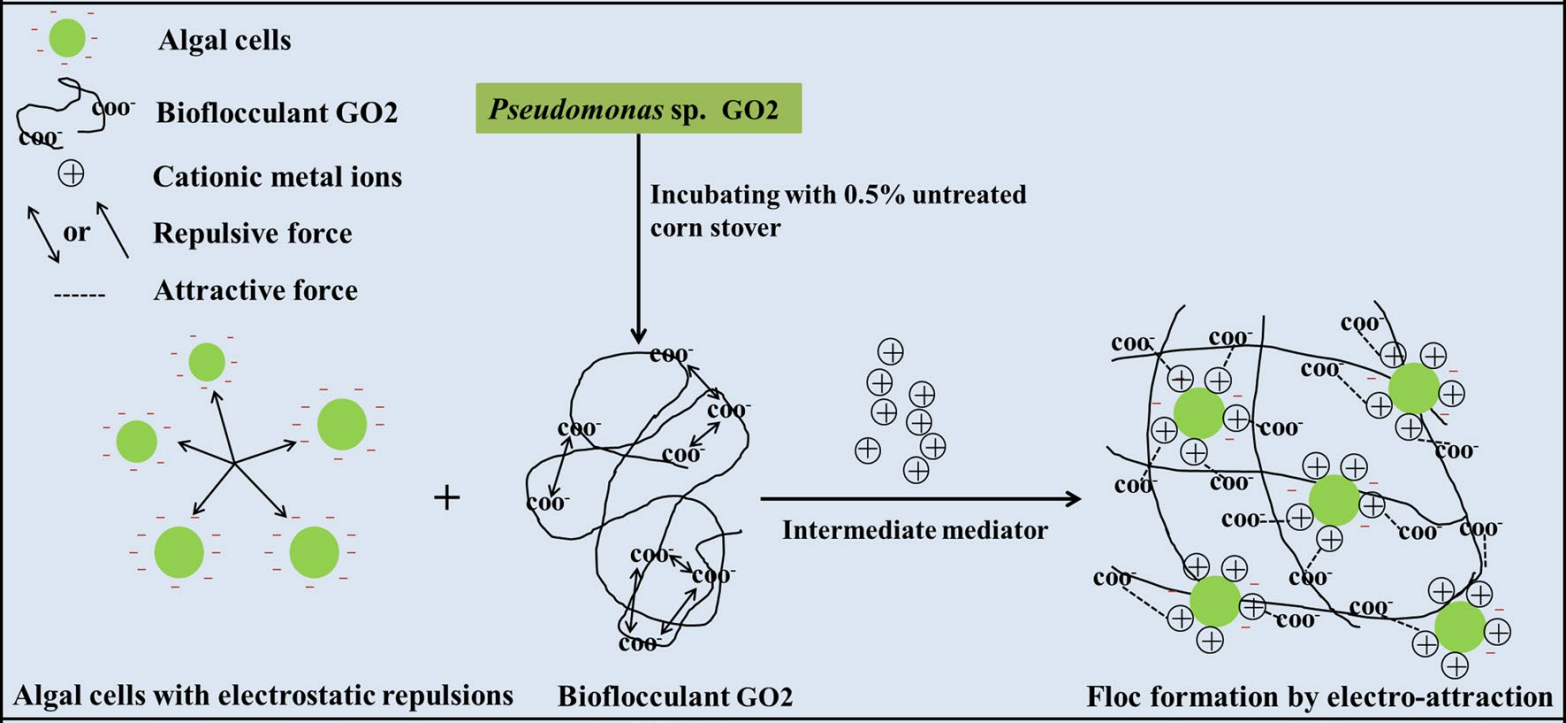
The possible mechanism of bioflocculants produced by GO2 in flocculating microalgal cells

Based on the characteristics analysis and the results of microalgae harvest, the possible flocculation mechanism of bioflocculants produced by GO2 was concluded as follows (Fig. 8): first, the bioflocculants produced by GO2 presented a negative zeta potential and charge density, which is the same with microalgae [14], indicating that microalgal harvest mechanism through these bioflocculants depended on sweeping and bridging, rather than electrical neutralization [52]; second, the cations were indispensable in the process of flocculation, further indicating bridging and patching played major roles in this flocculation. The cations can stabilize and neutralize the negative change of functional groups, which helped to overcome the electrostatic repulsion between bioflocculants and microalgal cells, and enhance flocculation; third, the high molecular weight bioflocculants can also stabilize the suspension and tend to flocculate through bridging. Bridging easily occurs when the extended distance of flocculant from the surface of particles into the solution is greater than the distance from the repulsion of interparticles [53].

## Conclusions

Low-cost bioflocculant was produced from untreated corn stover by a biomass-degrading bacterium *Pseudomonas* sp. GO2. The gram’s iodine staining and BRT analysis showed that GO2 directly utilized untreated biomass as carbon source to produce bioflocculant. An ideal flocculating efficiency of 99.8% was obtained under the optimal conditions (fermentation time 130.46 h, initial pH 7.46, and corn stover 0.64%). The extracted bioflocculant showed a high pH and temperature tolerance with a maximum flocculating efficiency of 94.7% at the dosage of 12.5 mg L^−1^. Furthermore, the low-cost bioflocculant can effectively harvest two green microalgae in a low GO2 fermentation broth/algal culture ratio.

## Methods

### Isolation and identification of bioflocculant-producing bacteria

A biomass-degrading bacterium was isolated from paper mill sludge by Gram’s iodine staining method according to our previous description [28]. When culturing the strain in the mineral salt medium (NaNO_3_ 0.1 g L^−1^, K_2_HPO_4_ 0.1 g L^−1^, KCl 0.1 g L^−1^, MgSO_4_ 0.05 g L^−1^, yeast extract 0.05 g L^−1^, and peptone 0.3 g L^−1^) containing 0.5% various untreated lignocellulosic biomasses, the fermentation broths became highly transparent and viscous, and biomass was compact and deposited at the bottom of flask after 48 h of incubation at 37 °C and 200 rpm. The strain was finally confirmed as a bioflocculant-producing bacterium by evaluating the flocculating rate of the fermentation broth. To identify the species of this bacterium, its genome was extracted using a Bacteria DNA kit (Bio Basic, Markham, Ontario, Canada). The 16S rRNA gene was amplified through polymerase chain reaction (PCR) using the primers: HAD-1 (5′-GACTCCTACGGGAGGCAGCAT-3′) and E1115R (5′-AGG GTTGCGCTCGTTGCGGG-3′). Then the PCR products were purified for sequencing.

### Evaluation of biomass degradation ability and enzyme activity assay

The biomass degradation ability of this strain was evaluated by using various lignocellulosic biomasses as carbon source as described by Guo et al. [28]. In brief, 5 μL of overnight-grown culture was inoculated on agar plates containing above mineral salt medium, 1.5% agar, and 0.5% agave, corn stover, *Miscanthus*, wheat bran, wood dust, CMC or beechwood xylan (Sigma-Aldrich, St. Louis, MO, USA). After incubation at 37 °C for 48 h, all of the plates were treated with Gram’s iodine reagent for 3–5 min. Then the diameters of the halo region (*D*) were measured on a centimeter scale.

The activities of CMCase and xylanase were evaluated by using CMC and xylan as substrate, respectively, as descripted by Guo et al. [28]. The concentrations of reducing sugar were determined using the 3,5-dinitrosalicylic acid (DNS, Sigma-Aldrich, St. Louis, MO, USA) reagent described by Miller [54]. A range of d-glucose and d-xylose (0–1.2 mg mL^−1^) concentrations were used to plot the standard curves for the hydrolysis production of CMC and xylan, respectively.

### Measurement of flocculating efficiency

The flocculating efficiency was tested as described by Kurane et al. [55] with some minor modifications. In brief, 200 μL of sample and 1.0 mL of 10% (w/v) CaCl_2_ were mixed with 40 mL of 0.5% (w/v) kaolin clay (Sigma-Aldrich, St. Louis, MO, USA) solution in a 50 mL glass-beaker, shaken at 100 rpm for 2 min, and left to stand for 1 min at 25 ± 2 °C. The optical density (OD) of the upper phase was measured at 550 nm using a microplate spectrophotometer (Epoch, Bio Tek Instruments, Inc., Vermont, USA). The flocculating efficiency was calculated as follows: Flocculating efficiency (%) = (*A* − *B*)/*A* × 100 × *D*, where *A* and *B* are the optical densities of the control and the samples at 550 nm, respectively, and *D* is the dilution time of the supernatant of the fermentation broth.

### Optimization of bioflocculant production and boosted regression tree (BRT) analysis

To estimate the effects of various biomasses on the production of bioflocculants using *Pseudomonas* sp. GO2, the strain was cultivated in the mineral salt medium containing 0.5% (w/v) untreated agave, corn stover, *Miscanthus* and wheat bran at 37 °C with agitation at 200 rpm, and the flocculating efficiency was monitored every day for 8 days. To determine the effects of incubation time, initial pH and biomass content on the production of bioflocculants, an experimental design matrix was performed via RSM using the Design-Expert software (Version 8.0.6., Stat-Ease Inc., Minneapolis, USA) according to our previous description [45]. The matrix included a total of 20 experiments, and all experiments were executed at 37 °C with shaking at 200 rpm. The fermentation broths were harvested every day and centrifuged at 12,000*g* for 3 min. Then the supernatant was used to determine the flocculating efficiency, and CMCase and xylanase activities.

To partition independent influences of incubation time, CMCase activity, xylanase activity, biomass concentration, and initial pH on bioflocculants’ production, The BRT model was built by the R-software (version 2.10.1, R development Core team 2009) with the ‘*gbm*’ package using a Bernoulli error structure [56], and with *brt.functions* [39]. The relative influence of each parameter in the model was measured according to the number of times that variable was selected for splitting in the tree, weighted by the squared improvement and averaged over all trees [57].

### Extraction and characteristics of the bioflocculants

The bioflocculants produced by GO2 were purified as descripted by Xiong et al. [58] with some minor modifications. In brief, GO2 strain was cultured using untreated corn stover as carbon source to produce bioflocculants in the optimal conditions. Then the fermentation broth was stored at 4 °C for at least 6 h to settle the solids. The supernatants were carefully transferred to a new flask and mixed with two volumes of pre-cooling ethanol, and the resultant sediment was collected by centrifugation at 5000*g* for 10 min, washed three times with 75% ethanol, and finally lyophilized to acquire the dryness bioflocculants.

To analyze the compositions of bioflocculants, the total polysaccharides were determined using the anthrone-sulfuric acid method, and d-glucose was selected as the standard sample [59]; the total protein was obtained by using the Bradford Protein Assay Kit (Bio Basic Canada Inc., Markhan, ON, CA) according to our previous description [60]; the uronic acid content was measured by cabazoic sulfuric acid method with d-(+)galacturonic acid as the standard sample [61]; while the nucleic acid content was quantified by the spectrophotometer. The molecular weight, charge density and Zeta potential of the bioflocculants were determined as descripted by Wang et al. [62]. The viscosity of the bioflocculants was measured using an OB-C218 Ubbelohde Viscometer (Cannon instrument company, USA) according to the method of Cui et al. [63]. The spectrum of bioflocculant was measured by a Bruker Tensor 37 FTIR Spectrophotometer (Bruker Optics, Inc., Billerica, MA) according to our previous description [45].

### Flocculating properties of the extracted bioflocculant

To understand the flocculating properties of bioflocculants, the effects of bioflocculants dosage, pH, temperature, and various metal ions on the flocculating efficiency of kaolin clay solution were recorded. The dosage of bioflocculants was set to range from 1.25 to 18.75 mg L^−1^. For the measurement of optimal pH and temperature, the flocculating efficiencies were assayed at various pH values (3–13) and various temperatures (4–80 °C). To investigate the effects of various metal ions, the monovalent cations (Na^+^ and K^+^), bivalent cations (Ca^2+^ and Mg^2+^), and trivalent cations (Al^3+^ and Fe^3+^) at the concentrations of 2.5, 5.0, 7.5, 10.0, and 15.0 mg L^−1^ were used.

### Evaluation the effect of the bioflocculant on the harvest of microalgae

Two green microalgal strains *C. zofingiensis* and *N. oleoabundans* were obtained from Dr. Lu at Hubei University of Technology and Dr. Chirs Lan at the University of Ottawa, respectively. The cells were grown in BG-11 medium under the conditions of the room temperature (25 ± 1 °C) with a light/dark cycle of 16:8 h (cool-white fluorescent light intensity of 100 ± 2 μmol m^−2^ s^−1^), and a constant shaking at 150 rpm. After 2 weeks of incubation, the biomass yields of *C. zofingiensis* and *N. oleoabundans* were about 1.8 and 2.0 g L^−1^ dry weight, respectively, and the algal culture was used for the harvest experiment.

The flocculating efficiencies of the two green microalgae (*C. zofingiensis* and *N. oleoabundans*) were evaluated by directly incubating the GO2 fermentation broth with algal culture. Different volumes of the fermentation broth (0.5–4 mL) were mixed with 40 mL algal culture containing 1 mL 10% CaCl_2_ solution. The mixture was stirred at 100 rpm for 2 min, and allowed to settle for 10 min at room temperature. Then the OD of the upper phase was measured at 680 nm using a microplate spectrophotometer (Epoch, Bio Tek Instruments, Inc., Vermont, USA). The flocculating efficiency of the microalgae was calculated as follows: flocculating efficiency (%) = (*A* − *B*)/*A* × 100 × *D*, where *A* and *B* are the optical densities of the control and the samples at 680 nm, respectively; *D* is the dilution time of the supernatant of the fermentation broth.

The microscopic images of *N. oleoabundans* cells before, and after flocculation using the GO2 fermentation broth were observed using an Olympus BX51 microscope (Olympus Optical, Tokyo, Japan) as descripted by Guo et al. [28]. In brief, the cells were harvested by centrifugation at 4000*g* for 5 min and resuspended in equal volume of deionized water after washing three times with deionized water. Then 20 μL of appropriate diluted algae cells were attached to a clean glass slide, and picture was taken.

### Statistical analysis

All the experiments were analyzed in quadruplicate, and the data are shown as mean ± SD. Statistical analysis was performed by one-way analysis of variance using the program, SPSS (SPSS Inc., USA, version 13.0). The matrix plot of the halo diameter was performed by means of the Past software (version 3.16) [64].

## Additional file

**Additional file 1: Fig. S1.** Phylogenetic tree of *Pseudomonas* sp. GO2. 16S rRNA gene sequences were retrieved by BLAST searches in NCBI and subjected to phylogenetic analysis using the neighbor-joining method with MEGA6 using 1000 bootstraps. **Fig. S2.** Fourier transform infrared spectrum of the bioflocculants produced by *Pseudomonas* sp. GO2 strain.

## Abbreviations

BRT: boosted regression tree
CMC: carboxymethyl cellulose
CMCase: carboxymethyl cellulase
DNS: 3,5-dinitrosalicylic acid
RSM: response surface methodology
