# Supplementary material for: Bioflocculants’ production in a biomass-degrading bacterium using untreated corn stover as carbon source and use of bioflocculants for microalgae harvest
Source: Biotechnol Biofuels. 2017 Dec 20;10:306. doi: 10.1186/s13068-017-0987-6 (PMC5738095; doi:10.1186/s13068-017-0987-6)
Supplement: Supplementary file 1 — Additional file 1: Fig. S1. Phylogenetic tree of Pseudomonas sp. GO2. 16S rRNA gene sequences were retrieved by BLAST searches in NCBI and subjected to phylogenetic analysis using the neighbor-joining method with MEGA6 using 1000 bootstraps. Fig. S2. Fourier transform infrared spectrum of the bioflocculants produced by Pseudomonas sp. GO2 strain. [file 13068_2017_987_MOESM1_ESM.docx]

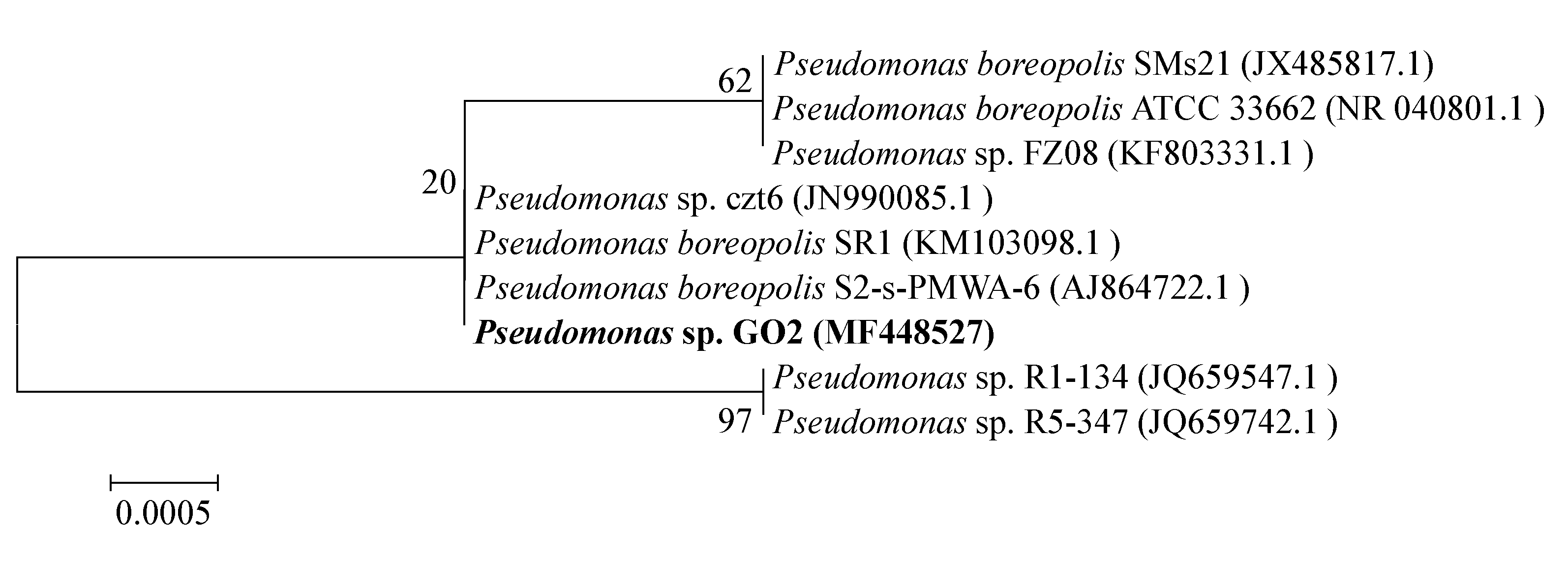


**Fig. S1** Phylogenetic tree of *Pseudomonas* sp. GO2. 16S rDNA gene sequences were retrieved by BLAST searches in NCBI and subjected to phylogenetic analysis using the neighbor-joining method with MEGA6 using 1,000 bootstraps.


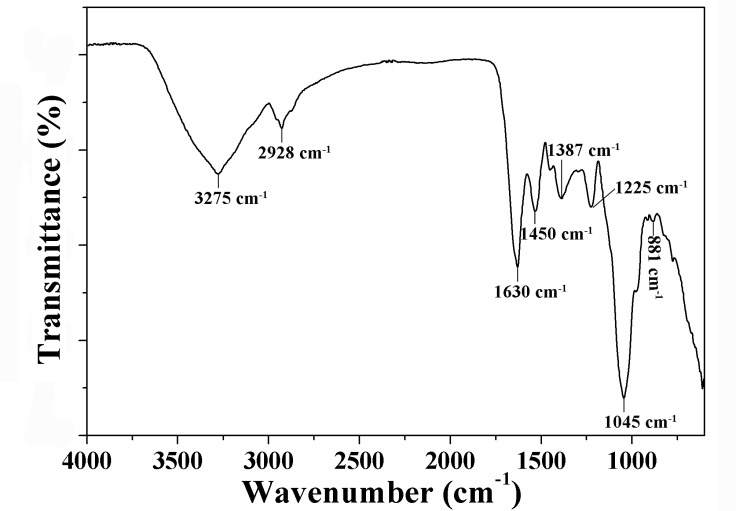


**Fig. S2** Fourier transform infrared spectrum of the bioflocculants produced by *Pseudomonas* sp. GO2 strain.
